# Simulation and Experimental Verification of Die Quenching Deformation of Aviation Carburized Face Gear

**DOI:** 10.3390/ma16020690

**Published:** 2023-01-10

**Authors:** Huaming Liu, Jiuyue Zhao, Jinyuan Tang, Wen Shao, Beier Sun

**Affiliations:** 1 State Key Laboratory of High Performance Complex Manufacturing, Central South University, Changsha 410017, China; 2 College of Mechanical and Electrical Engineering, Central South University, Changsha 410017, China

**Keywords:** die quenching, deformation, face gear, carburizing-quenching

## Abstract

The tooth width and length of face gear limit control the strength of face gear, and heat treatments are often used to improve the hardness and strength of face gear. However, heat treatments will often cause additional deformations, which will affect the dimensional accuracy of the face gear. In this paper, to effectively control the deformation and ensure the accuracy of the face gear, the finite element method was used to establish the calculation model of the face gear die quenching method, and thus, the influence of die on the gear quenching deformation was analyzed. Next, the accuracy of the calculation model was verified by the pressure quenching experiment. The results demonstrated that the inconsistent phase transformation between the surface and the center of the face gear was the key factor affecting the deformation due to the influence of the carbon content. Compared with die-less quenching, the inner hole-die can effectively limit the radial shrinkage deformation of the face gear. With the increase of the upper-die pressure, the axial and radial deformations of the face gear gradually became stable. In the actual production, the load of dies should be reasonably selected based on the gear accuracy requirements.

## 1. Introduction

With the characteristics of light weight, low vibration and noise, high coincidence, and insensitivity to axial errors, face gears are widely used in helicopter transmission systems, robot drives, and automotive fields [1]. Since the shape of face gear is different in the direction of the tooth width, and is affected by the root cutting and the sharp angle, the length of the tooth width of the face gear cannot be too long; therefore, the transmission strength of the gear is limited. The material properties and bearing capacity of gear can be improved by the heat treatment process. However, plastic strain is generated due to thermal stress and structural stress, which causes gear deformation. Larger deformation not only affects the gear accuracy, but also increases difficulties for subsequent grinding [2].

Quenching deformation is mainly affected by factors such as workpiece shape, material properties, and heat treatment process parameters. The corresponding optimization of the influencing factors can effectively reduce the additional deformation caused by quenching. The quenching medium determines the quenching cooling rate and is the key to determining what affects the deformation. AMEY et al. [3] studied the effects of water quenching, oil quenching, and salt baths on the quenching deformation of 100Cr6 steel through experiments. The results showed that salt baths can greatly reduce the deformation. In addition, alloying can balance the expansion deformation caused by bainite with the contraction deformation caused by the formation of residual austenite. Kim et al. [4] studied the effect of the quenching medium temperature on gear deformation. As the temperature of the quenching medium increases, the gear deformation decreases. Wang et al. [5] optimized the heat treatment process route, reducing the quenching temperature to effectively reduce the axial and radial deformations of the face gear. Immersion orientations would also affect the quenching deformations of the workpieces, where the deformation of the larger end immersion is smaller than that of the smaller end immersion, which is consistent with the orientation effect observed in actual production [6,7]. Dybowski et al. [8] optimized the heat treatment process by changing the quenching method. High-pressure gas quenching is more uniform in deformation than oil quenching, and oil quenching is suitable for steels with low hardenability. Heuer et al. [9] used low-pressure carburizing and high-pressure gas quenching technologies to heat-treat the workpiece with small and stable deformations. Husson et al. [10] found that the geometric shape of the part and the initial residual stress distribution were important factors affecting the heat treatment deformation of the workpiece. Pang et al. [11] analyzed the influence of workpiece structures on quenching deformation, and found that the distortion of the common normal of the solid shaft was greater than the distortion of the common normal of the hollow shaft. Brinksmeier et al. [12] analyzed the influence of forging and cutting the process parameters before the heat treatment on gear deformations through experiments, and reduced the carburizing and quenching deformation of gears starting from pre-treatment. Cho et al. [13] studied the effects of carburizing and quenching on the deformation of forged and machined gears. The average deformation of forged gears was greater than that of machined gears. In addition, many scholars had reduced the heat treatment deformation of the workpiece by the induction quenching [14,15].

Besides the above quenching deformation control methods, it is more effective to use die quenching to control deformation [16]. Dies limit the deformation of the gear during quenching to ensure that the deformation size is within the required range, and the internal stress caused by the forced anti-distortion can be eliminated by tempering. In the process of die quenching, the gear deformation is affected by the press pressure, quenching medium, carbon content, die, and so on. Improper quenching parameters may lead to larger deformations of the gear than in ordinary quenching, which would accelerate gear wear [17]. Li et al. [18] studied the die quenching deformation of spiral bevel gears through the professional heat treatment numerical calculation software, DANTE. Restricted by the expander, the deformation of the upper hole was reduced, and the deformation of the lower hole was increased. Increasing the size of the expander could effectively reduce the deformation. Based on the deformation trend of the upper hole and the lower hole, the shape of the expander was changed to a conical shape. The calculation results demonstrated that the deformation of the inner hole under the conical plug was more uniform, which solved the problem of the large radial shrinkage deformation of the spline during the quenching process. Zhang et al. [19] established a numerical model for the die quenching of bevel gears and analyzed the influence of hardenability on the deformation of bevel gears. The core of the high hardenability gear had more bainite than that of the low hardenability gear, so the inner hole deformation of the high hardenability gear was larger. Reardon [20] studied the quenching deformation of automobile gears. The results showed that the pretreatment had a great influence on the deformation of die quenching gear, and the out-of-round deformation of die quenching gear decreased with the increase of normalizing temperature. Knauf S. et al. [21] combined induction hardening with die hardening to achieve high-precision gearbox fabrication.

Due to the complexity of the face gear structure, there are a few studies on the die quenching of face gear. The influencing factors and final deformation trend of face gear during die quenching process are still unclear. It takes much time and economy to determine the process parameters of die quenching through experiments. Therefore, it is necessary to establish the finite element analysis model of die quenching, clarify the relationship between process parameters and gear deformation, and optimize the heat treatment process route. In this paper, based on the multi-field coupling theory of heat treatment, a method for constructing the finite element calculation model of face gear die quenching was proposed. By analyzing the hardened layer, temperature field, and microstructure field of the heat-treated gear, the correlation law among phase transformation, dies, press loads, and gear deformation during quenching process was clarified. The accuracy of the model was verified by the die quenching experiment, which provided theoretical guidance for the selection of die quenching parameters of the face gear.

## 2. Finite Element Theory and Modeling of Die Quenching

### 2.1. Modeling of Face Gear

In this paper, based on the gear shaping process of a straight gear cutter, a three-dimensional model of the face gear was built in CAD software. The normal modulus of a face gear was 3.9, while the number of teeth was 142, and the normal pressure angle was 25°. An involute gear shaper cutter was used to process the face gear. In the modeling of face gear, the shaper could be simplified as a spur gear meshing with face gear, and the meshing process was the tooth surface generation process of face gear. The rotation angle of face gear and spur gear changed with a fixed transmission ratio:

(1) $$\frac{\omega_{2}}{\omega_{s}}=\frac{\varphi_{2}}{\varphi_{s}}=\frac{N_{s}}{N_{2}}$$

where $\varphi_{2}$ and $\varphi_{s}$ are the rotation angles of the face gear and spur gear. $\omega_{2}$ and $\omega_{s}$ are the angular velocities of the face gear and spur gear. $N_{2}$ and $N_{s}$ are the number of teeth of the face gear and spur gear. The coordinate system $S_{2}$ of the face gear and the coordinate system $S_{s}$ of the spur gear were built in Catia. The coordinate origin was located at the intersection of the shaper axis and the face gear axis, where the axis $y_{2}$ was the symmetry line of the tooth groove, and the axis $x_{2}$ was determined according to the right-hand rule. The transformation matrix of the spur gear coordinate system and face gear coordinate system is related as [1]:

(2) $$M_{2s}=\left[\begin{array}{cccc} \cos\varphi_{s}\cdot \cos\varphi_{2}+\cos\gamma_{m}\cdot \sin\varphi_{s}\cdot \sin\varphi_{2} & -\sin\varphi_{s}\cdot \cos\varphi_{2}+\cos\gamma_{m}\cdot \cos\varphi_{s}\cdot \sin\varphi_{2} & -\sin\gamma_{m}\cdot \sin\varphi_{2} & 0\\ -\cos\varphi_{s}\cdot \sin\varphi_{2}+\cos\gamma_{m}\cdot \sin\varphi_{s}\cdot \cos\varphi_{2} & \sin\varphi_{s}\cdot \sin\varphi_{2}+\cos\gamma_{m}\cdot \cos\varphi_{s}\cdot \cos\varphi_{2} & -\sin\gamma_{m}\cdot \cos\varphi_{2} & 0\\ \sin\gamma_{m}\cdot \sin\varphi_{s} & \sin\gamma_{m}\cdot \cos\varphi_{s} & \cos\gamma_{m} & 0\\ 0 & 0 & 0 & 1 \end{array}\right]$$

The tooth surface of the face gear can be divided into the working part and fillet part. Based on the meshing principle, the projection of the relative velocity vector of the tooth surface at the meshing point on the common normal was zero. The tool surface was $r_{s}\left(\mu_{r},\mu_{z}\right)$, and the meshing point was transformed through the transformation matrix $M_{2s}$. The equation of the working part can be obtained as [22]:

(3) $$\{\begin{array}{c} \left[\begin{array}{c} r_{2w}\left(\mu_{r},\mu_{z},\varphi_{s}\right)\\ 1 \end{array}\right]=M_{2s}\left(\varphi_{s}\right)\cdot \left[\begin{array}{c} r_{s}\left(\mu_{r},\mu_{z}\right)\\ 1 \end{array}\right]\\ f_{2}\left(\mu_{r},\mu_{z},\varphi_{s}\right)=N_{2}\left(\mu_{r},\varphi_{s}\right)\cdot V_{2}^{\left(2s\right)}\left(\mu_{r},\mu_{z},\varphi_{s}\right)=0 \end{array}$$

where $V_{2}^{\left(2s\right)}$ is the velocity of the shaper at the meshing point relative to the face gear in the spur gear coordinate system. The fillet part was the sweep surface formed by the spur gear cutter tooth tip. The calculation formula is as follows:

(4) $$\left[\begin{array}{c} r_{2f}\left(\mu_{z},\varphi_{s}\right)\\ 1 \end{array}\right]=M_{2s}\left(\varphi_{s}\right)\cdot \left[\begin{array}{c} {r_{s}\left(\mu_{r},\mu_{z}\right)|}_{\mu_{r}=\mu_{top}}\\ 1 \end{array}\right]$$

**Figure 1:**
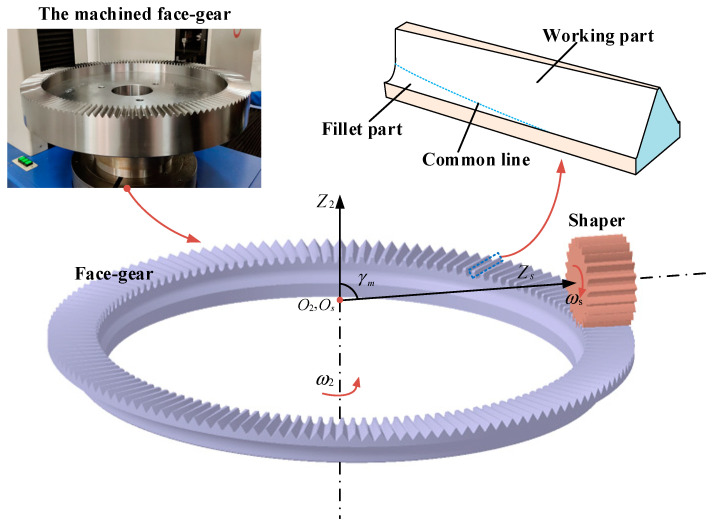
Modeling Process of Face Gear Machining by Spur Gear Tool.

The common line was the locus of the top point of the spur gear tooth. The points on the common line conformed to the meshing equation. The modeling process of face gear is shown in Figure 1.

### 2.2. Numerical Theoretical Model for Heat Treatment

Carburizing-quenching is a complex physical process. The level of carbon potential content and temperature change directly affect the composition and transformation content of the structure. During the quenching process, the changes of carbon potential and temperature directly affect the composition and transformation content of the structure, the specific heat capacity and thermal conductivity change dynamically with the content of the structure, and the latent heat released by the phase transformation affects the change of the temperature field. The specific tolerance between the parent phase and the daughter phase during the phase transformation produces tissue stress, and the phase transformation products affect the mechanical properties of the material. Thus, heat treatment is a mutual coupling process of carburizing field, temperature field, microstructure field, and stress-strain field [23].

#### 2.2.1. Carbon Diffusion Model

Carburizing is mostly completed by heating the part in a pit furnace or sealed atmosphere furnace and introducing carburizing gas at high temperatures. Carburizing increases the surface strength and wear resistance by diffusing carbon atoms into the gear steel, while maintaining toughness and strength at the core. The distribution of carburizing concentration field conforms to Fick’s second law:

(5) $$\frac{\partial C}{\partial t}=D\left(\frac{\partial^{2}C}{\partial x^{2}}+\frac{\partial^{2}C}{\partial y^{2}}+\frac{\partial^{2}C}{\partial z^{2}}\right)$$

where t is time in seconds, C is the carbon concentration as a function of displacement and time in the direction of diffusion, and D is the diffusion coefficient in $m^{2}/s$, which increases with the increase of temperature and carbon content [24]. The carbon diffusion coefficient is affected by the alloy composition, and the calculation formula can be modified as [25]:

(6) $$D=0.0047\exp\left(-1.6C\right)\exp\left(\frac{-\left(37000-6600C\right)}{RT}\right)q$$

where q is the constant related to the alloy composition and R is the ideal gas constant with a value of 8.314 J/mol/K.

#### 2.2.2. Temperature Calculation Model

Assuming that the material is isotropic, the thermal conductivity in the x, y, and z directions is the same. Considering the latent heat of phase transformation, the heat conduction equation in three-dimensional space can be derived using the principle of energy conservation [26]:

(7) $$\lambda \left(\frac{\partial^{2}T}{\partial x^{2}}+\frac{\partial^{2}T}{\partial y^{2}}+\frac{\partial^{2}T}{\partial z^{2}}\right)+q_{v}=\rho c_{p}\frac{\partial T}{\partial t}$$

where λ is the thermal conductivity in W/m/K, $q_{v}$ is the latent heat of phase transformation and the heat of plastic work formation, and the heat of plastic work formation is generally 2~3 °C, which can be ignored [27]. ρ is the material density in $kg/m^{3}$ and $c_{p}$ is the specific constant pressure heat capacity in J/kg/K. The surface of the part exchanges heat with the external fluid medium during quenching, which is a convection boundary condition [28]:

(8) $$-\lambda {\frac{\partial T}{\partial n}|}_{s}=H_{k}\left(T-T_{f}\right)$$

where $H_{k}$ is the heat transfer coefficient in $W/m^{2}/K$ and $T_{f}$ is the medium temperature in Kelvin.

#### 2.2.3. Kinetic Model of Phase Transformation

Various structural transformations will occur according to the cooling rate and the material properties of the workpiece during the quenching process. The transformation from austenite to ferrite, and pearlite and bainite belongs to the diffusive phase transformation, while the transformation from austenite to martensite belongs to non-diffusive phase transformation. Based on the grain nucleation theory, the Johnson-Mehl-Avrami-Kolmogorov (JMAK) equation is often used to calculate diffusive phase transformation equations at constant temperature [29]:

(9) $$\phi =1-\exp\left(-bt^{n}\right)$$

where ϕ is the transformation amount, t is the isothermal time in second, and b and n are constants on the TTT curve of the material. The JMAK equation describes the transformation process under isothermal conditions, which cannot be used directly for continuous cooling. The Scheil rule is used to discretize time and simulate multi-stage isothermal phase transformations in the continuous cooling process [30]. Many scholars have shown that the Scheil rule has a large error in its calculation and experiments [31]. With the development of thermal compression experiments, differential form phase variable computations are developed [32]:

(10) $$\frac{d\phi_{d}}{dt}=v_{d}\left(T\right)\phi_{d}^{\alpha_{1}}{\left(1-\phi_{d}\right)}^{\beta_{1}}\phi_{a}$$

where $\phi_{d}$ is the content of the new phase structure, $\phi_{a}$ is the volume fraction of austenite, $v_{d}\left(T\right)$ is the diffusion phase mobility related to the expansion curve, and $\alpha_{1}$ and $\beta_{1}$ are the kinetic constants of the diffusion phase transformation related to the carbon content. Calculation by the dilatometric curve [32] and the kinetic constants of 9310 material are shown in Table 1.

The transformation amount of non-diffusive phase transformations only depends on temperature change and is independent of time. The K-M equation is often used to calculate the transformation amount of martensite for low carbon steel [33]:

(11) $$V=1-\exp\left[-\alpha \left(M_{s}-T\right)\right]$$

where $M_{s}$ is the starting temperature of the martensitic transformation in Kelvin and α is the kinetic constant of the martensitic transformation rate, which depends on the material properties. Other scholars have found that the K-M equation is suitable for burst-like transformations, and cannot reflect the sigmoid function of the martensite volume fraction as a temperature. A new kinetic equation for martensite transformation is proposed [34]:

(12) $$\frac{d\phi_{M}}{dT}=v_{m}{\left(1-\phi_{M}\right)}^{\alpha_{2}}{\left(\phi_{M}+\varphi \phi_{d}\right)}^{\beta_{2}}\phi_{a}$$

where $\phi_{M}$ is the volume fraction of martensite, $v_{m}$ is the mobility of martensite, φ, $\alpha_{2}$, and $\beta_{2}$ are the kinetic constants of martensite transformations, and T is the temperature during quenching in Kelvin. Based on the dilatometric curve, the values of each parameter of 9310 steel are shown in Table 2.

#### 2.2.4. Mechanical Model

Using the theory of small deformation, the full strain increment for heat treatment is decomposed into five parts [35]:

(13) $$d\varepsilon =d\varepsilon^{e}+d\varepsilon^{p}+d\varepsilon^{t}+d\varepsilon^{tr}+d\varepsilon^{tp}$$

where $\varepsilon^{e}$ is the elastic strain increment, $\varepsilon^{p}$ is the plastic strain increment, $\varepsilon^{t}$ is the temperature strain increment, $\varepsilon^{tr}$ is the train increment caused by phase transformation, and $\varepsilon^{tp}$ is the strain increment caused by phase transformation plasticity. $\varepsilon^{e}$ is calculated by Hooke’s law, considering the influence of temperature and phase transformation field on Jacobian matrix. $\varepsilon^{t}$ is calculated from the coefficient of thermal expansion determined by the thermal expansion curve. The temperature causes austenite to undergo diffusive and non-diffusive transformations. When atoms are not tightly packed in the lattice, $\varepsilon^{tr}$ is calculated by the following formula:

(14) $$d\varepsilon_{ij}^{tr}=\sum_{k=1}^{p}\frac{1}{3}\Delta_{k}\Delta \xi_{k}\delta_{ij}$$

where $\Delta \xi_{k}$ represents the phase change increment and $\Delta_{k}$ represents the structural expansion caused by the decomposition of austenite into ferrite, pearlite, bainite, and martensite. In the quenching phase transformation process, transformation-induced plasticity (TRIP) significantly increases plasticity, and the plastic deformation also occurs when the equivalent stress of the applied load is lower than the yield strength of the parent phase [36]. Greenwood and Johnson [37] have proposed a plastic calculation model for phase transformation, considering the specific volume change and the yield strength of the weak phase in the process of phase transformation. Abrassart [38] further considered the effect of austenite decomposition on transformation plasticity and modified the transformation plasticity:

(15) $$d\varepsilon_{ij}^{tp}=\frac{9}{2}k\left(1-\sqrt{\xi }\right)\dot{\xi }S_{ij}$$

where $S_{ij}$ is the deviant stress, ξ is the structural content of the new phase, and $\dot{\xi }$ is the rate of change of the new phase with time.

Desalos considered the effect of complex stress, and proposed a transformation plasticity function ϕ(ξ) that can be used for diffusion-type transformation and martensitic transformation. The relationship between phase transformation plasticity and stress and phase variable can be described as [28]:

(16) $$d{\dot{\varepsilon }}_{ij}^{tp}=3k\left(1-\xi \right)\dot{\xi }S_{ij}$$

where ${\dot{\varepsilon }}_{ij}^{tp}$ is the rate of change of phase transformation plasticity with time.

### 2.3. Material Properties of 9310

**Figure 2:**
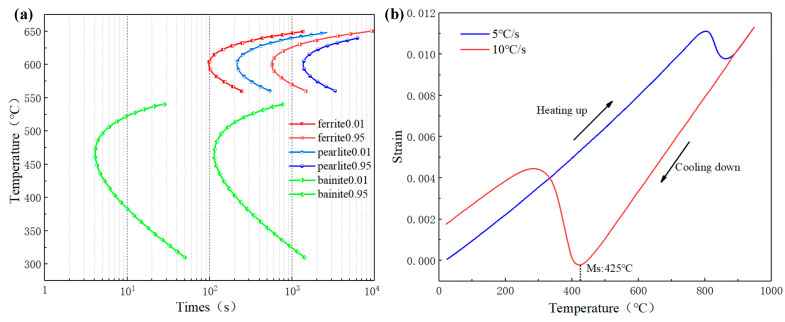
Phase transformation kinetic parameters of 9310 with 0.1% carbon content: (**a**) TTT diagram; (**b**) Dilatometric curve.

The material of face gear is 9310 steel, which has a high hardenability, high hardness, and high fatigue strength. It is mainly used in aviation gear manufacturing. Table 3 shows the chemical composition of 9310 steel. In the process of the heat treatment calculation, the TTT curve is the basis of the microstructure evolution calculation, which reflects the relationship between isothermal time and microstructure transformation of supercooled austenite at different temperatures. The TTT diagram of 9310 was calculated by DANTE, as shown in Figure 2a. The incubation period of ferrite and pearlite is longer than that of bainite. Based on the faster cooling rate of quenching, ferrite and pearlite will not be generated in the gear. The phase transformation process is accompanied by a volume change, and the beginning temperature of the phase transformation can be determined by the dilatometric curve. When the volume increases from the austenite face-centered cubic lattice to the martensite body-centered cubic lattice, the dilatometric curve will change abruptly. The temperature of the abrupt change point corresponds to the martensite start temperature ($M_{s}$). When the heating rate is 5 °C/s, the austenitizing temperature is maintained for 30 min, and then cooled at 10 °C/s. The dilatometric curve was calculated by DANTE as shown in Figure 2b, where the $M_{S}$ temperature is 425 °C.

**Figure 3:**
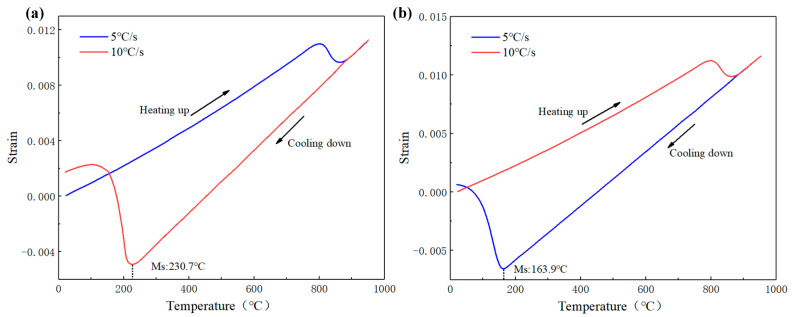
Dilatometric curves of different carbon content: (**a**) 0.5% carbon; (**b**) 0.8% carbon.

After gear carburization, the surface carbon content affects the austenite lattice shear and increases the martensite transformation resistance. Many scholars have fitted the martensite start temperature through dilatometric experiments [39]. The thermal simulation analysis of cylindrical samples was carried out by DANTE. The dilatometric curves of different carbon contents are shown in Figure 3. The $M_{S}$ temperature of 0.5% carbon content is 230.7 °C, while the $M_{S}$ temperature of 0.8% carbon content is 163.9 °C. With the increase of the carbon content, the $M_{S}$ temperature decreases gradually.

**Figure 4:**
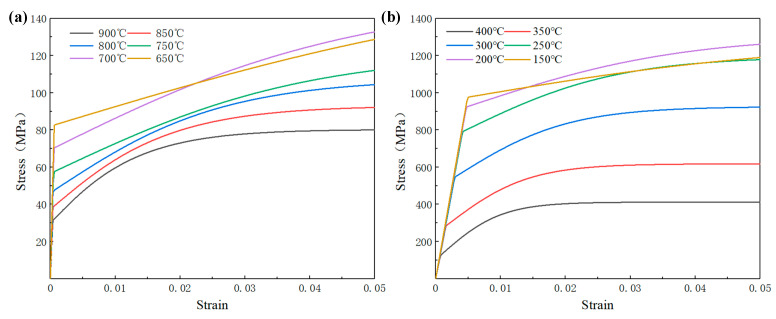
True stress-strain curves of 9310 steel at different temperatures: (**a**) austenite; (**b**) martensite.

The austenite-martensite transformation during the quenching process, the thermal conductivity, and the specific heat capacity of different phases are shown in Table 4. The maximum strain generated during heat treatment is only 2~3%. The austenite and martensite stress-strain curves corresponding to different temperatures at a strain rate of 0.01 were calculated by DANTE. Figure 4 shows the stress-strain curve of austenite and martensite. The yield stress of austenite and martensite gradually increases as the temperature decreases, and the yield stress of martensite is far greater than the yield strength of austenite. The elastic modulus and plastic modulus can be determined by the stress-strain curve, and the final stress-strain curve of the material follows the linear superposition rule.

### 2.4. Finite Element Modeling of Die Quenching

#### 2.4.1. Parameters of the Die Quenching Model

**Figure 5:**
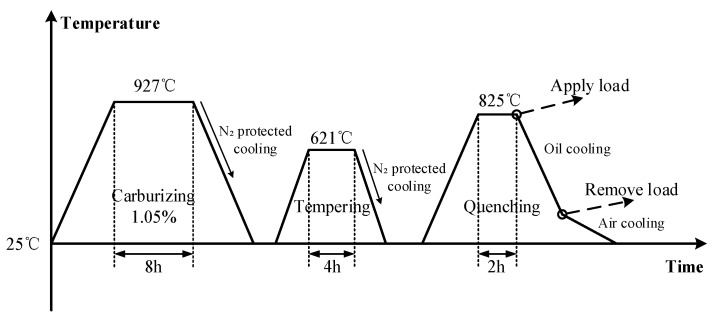
Process curve for the die quenching of the face gear.

**Figure 6:**
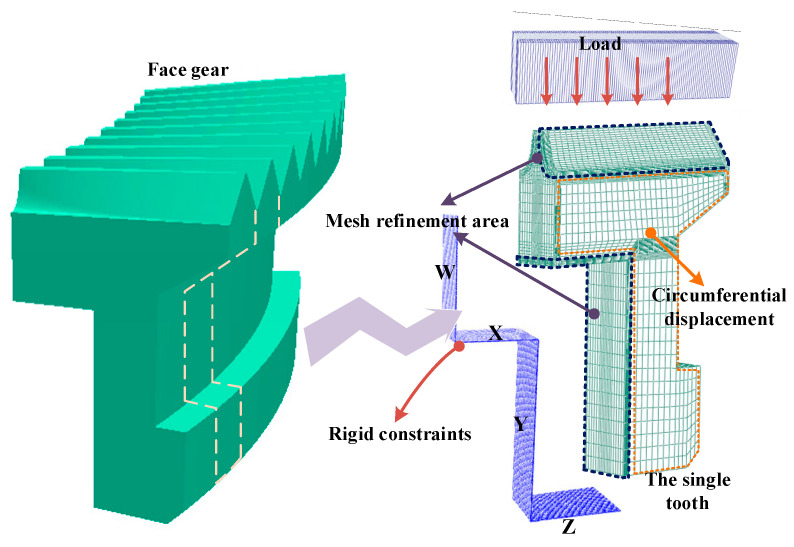
Geometry and mesh of the face gear model; boundary constraints for die quenching.

The heat treatment process route of the face gear is shown in Figure 5. The heat treatment process of the face gear included carburizing, high temperature tempering, and die quenching with the total time being 14 h. Due to the symmetry of the gear structure, 1/142 of the gear was selected for modeling to shorten the calculation time. In the simulation of carburizing and quenching, the calculation accuracy of carbon potential gradient and temperature gradient on the surface of the gear was affected by the mesh size, and it was necessary to refine the mesh on the surface of the gear. The finite element model was divided by hexahedral mesh, including 60,620 elements and 66,563 nodes. The mesh in the carburized area was locally refined, where the refinement layer depth was 1 mm, and the mesh thickness was 0.1 mm. The face gear was mainly constrained by the inner hole support ring W, X, Y, and the lower die Z in the die quenching process. Meanwhile the upper die applied a load of 71 KN vertically down to the top of the gear. The dies were set as a rigid body, where the dies and gear surface contacted with the surface during loading, and unloaded after quenching. The contact type between the die and the gear was surface to surface contact. To prevent penetration between contact pairs, the normal contact was hard contact. Considering the cyclic symmetric structure of the gear, a cylindrical coordinate system was established on the cyclic symmetric plane of the gear to limit the circumferential displacement and allow the gear to expand radially. Finite element meshing and boundary constraints of face gear are shown in Figure 6.

#### 2.4.2. Boundary Conditions for Quenching

**Figure 7:**
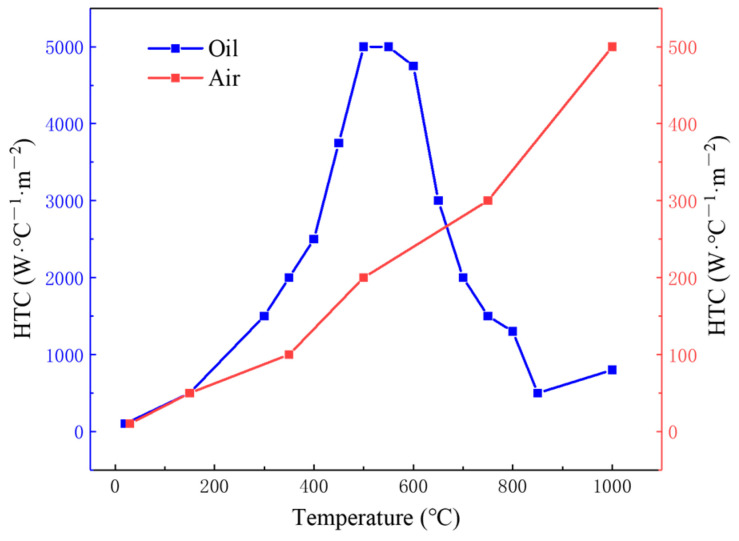
Heat transfer coefficient (HTC) of quenching oil and air cooling.

During quenching, the gear temperature and microstructure change drastically, and the heat transfer between the surface of the gear and quenching medium will progress through three processes: The first process is the vapor blanket stage. The surface of the gear is just in contact with the quenching medium, and the quenching medium will generate vapor to cover the surface of the gear to form the vapor blanket, which will separate the surface of the gear from the quenching medium resulting in a small heat transfer coefficient. The second process is the nucleate boiling stage. When the surface temperature of the gear decreases to a certain level, the vapor blanket covering the surface of the gear will rupture, and the surface of the gear will be in direct contact with the cooling medium resulting in a sharp increase in the heat transfer coefficient. The third process is the convective cooling stage. When the surface temperature of the part drops below the boiling point of the cooling medium, the nucleate boiling stage ends, the heat transfer coefficient decreases, and the cooling rate decreases [40]. The heat transfer coefficient directly affects the stress and deformation during the quenching process. Heat transfer coefficients of oil and air cooling in the finite element model were calculated by DANTE, as shown in Figure 7.

## 3. Results and Discussion

### 3.1. Deformation of Gear Die Quenching

During quenching, gear deformation is caused by uneven cooling and microstructure transformation. Therefore, it is very important to understand the temperature history and microstructure transformation for the analysis of heat treatment deformation in quenching process.

#### 3.1.1. Temperature History and Microstructure Transformation

**Figure 8:**
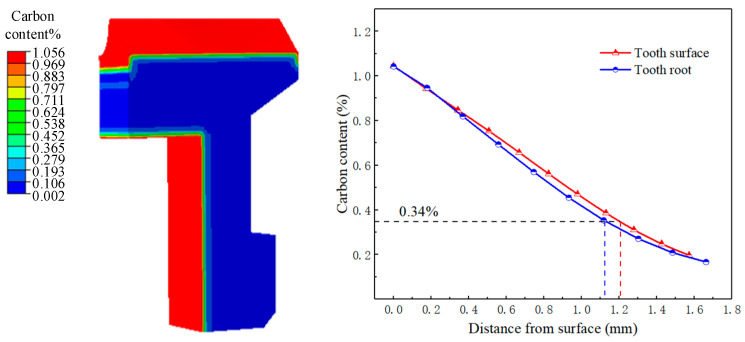
Carbon content distribution of the tooth surface and the tooth root.

The phase transformation temperature of the carburized gear is affected by the carbon content. The time of the phase transformation in each part of the gear can be determined by comparing the temperature history with the microstructure transformation. The carbon content distribution of the face gear after carburizing is shown in Figure 8. The carbon content of the tooth surface was 1.05%, and the carbon content of the core was 0.1%. With the increase of the depth from the surface, the carbon potential gradually decreased. The carbon content of the tooth surface was higher than that of the tooth root. With 0.34% carbon content as the standard, the depth of the tooth surface carburizing layer was about 1.2 mm, and the depth of the tooth root carburizing layer was 1.1 mm.

**Figure 9:**
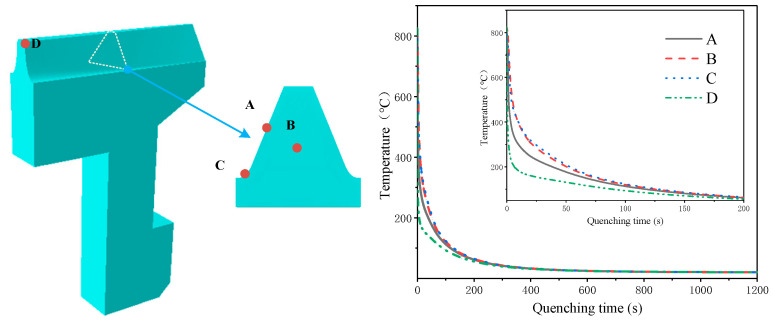
Temperature variation during gear quenching.

The specific locations of the points and the temperature changes during quenching are shown in Figure 9 (tooth surface: point A, core: point B, tooth root: point C, and tooth top: point D). The temperature of each point changed drastically within 200 s, and the temperature difference between inside and outside was essentially eliminated at around 400 s. The gear surface points A, C, and D were in direct contact with the quenching oil, and the temperature dropped rapidly. The temperature change rate of point D on the tooth top was the fastest, and the temperature change rate of point C at the root was the slowest. The temperature drop rate of point B was lower than that of point A due to the internal heat transfer of the gear. The temperature change curves of point B and point C were basically the same. The results showed that the temperature change was affected by the shape of the part, where the temperature at the top of the tooth changed most dramatically.

**Figure 10:**
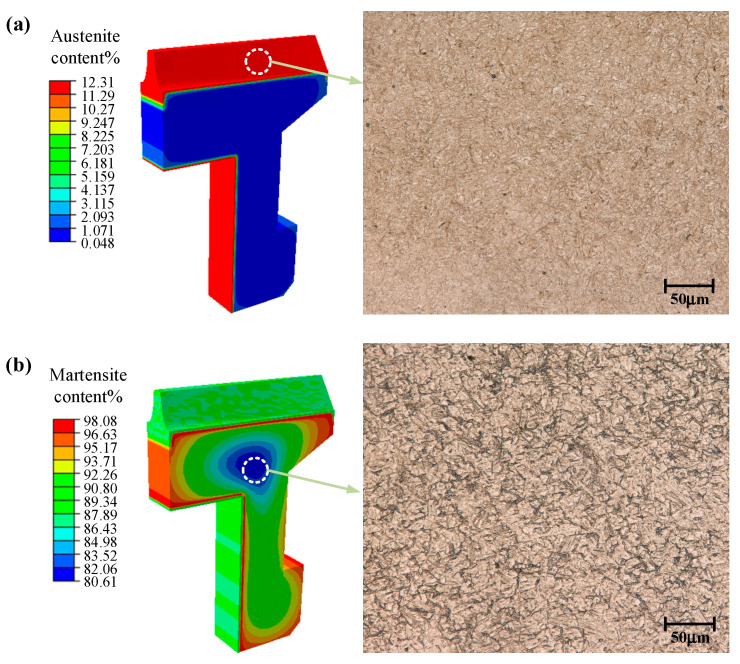
Distribution of metallographic structure for the face gear after the quenching process: (**a**) Austenite; (**b**) Martensite.

**Figure 11:**
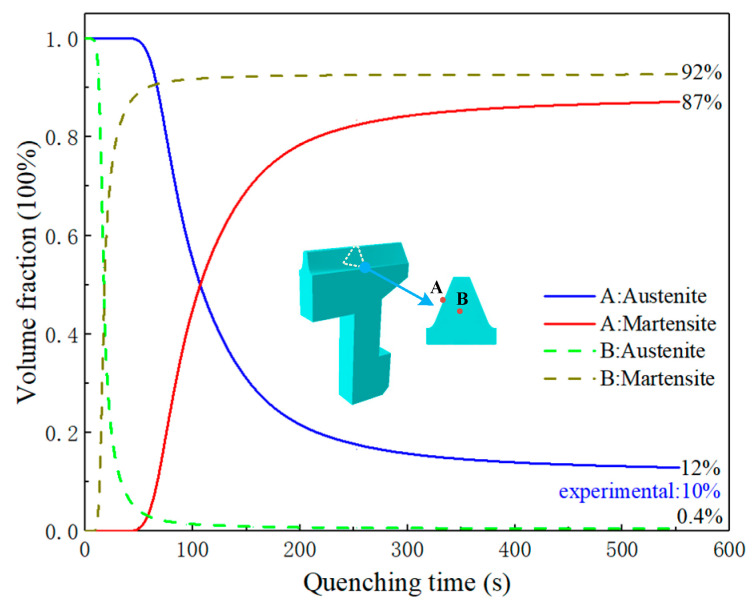
Phase transformation in the gear quenching process: A: tooth surface; B: tooth core.

After carburizing and quenching, the final structures of the gear were mainly martensite and austenite. The carbon content in austenite was increased by carburizing, and it was difficult for the carbon element to precipitate during quenching. At the same time, the $M_{s}$ temperature and the martensite transformation amount decreased with the increase of carbon content, therefore, the residual austenite was concentrated in the carburized areas of the gear surface. The metallographic polishing machine was used to grind and polish the face gear samples. After polishing, the samples were corroded with a 4% nitrate alcohol solution, and finally the microstructure was observed by an ultra-depth of field three-dimensional microscopic system (KeyenceVHX-5000). The residual austenite was grayish-white in the metallographic structure and lighter in color than the spiculate martensite. The core was mainly composed of martensite and a small amount of bainite, which turned black after being corroded. The main microstructure distribution of the gear after quenching is shown in Figure 10. In the quenching process, the position with a faster cooling rate was more likely to undergo martensite transformation, which may be changed by the influence of carbon content. Figure 11 shows the phase transformation history of the gear tooth surface and core. The $M_{s}$ temperature of the tooth surface was relatively low; therefore, the austenite began to transform into martensite at about 50 s, and the transformation was mostly completed at roughly 350 s. The cooling rate of the core was slow, but the $M_{s}$ temperature was higher than at the surface and the martensite transformation began at about 10 s, and the transformation was basically completed around at about 100 s. For the carburized gear, the martensite transformation began first in the core, and the phase transformation of the core was completed before that of the surface.

#### 3.1.2. Gear Deformation of Die Quenching

**Figure 12:**
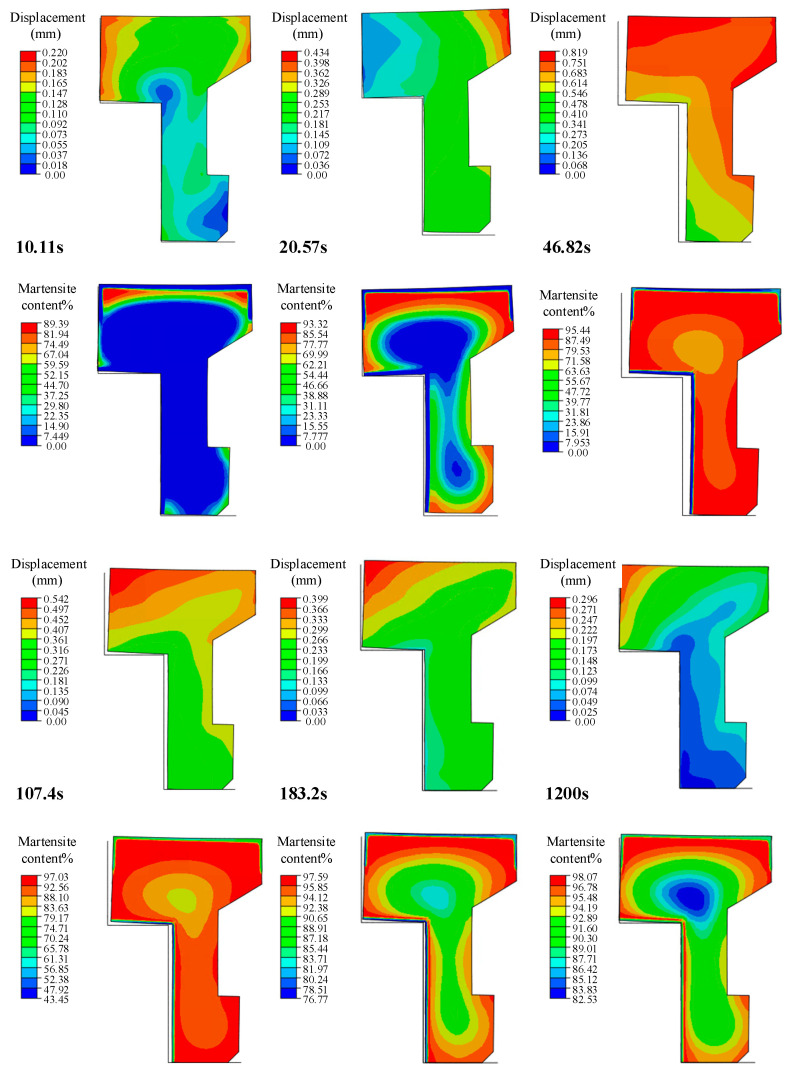
Gear deformation and martensitic transformation at critical time points.

In the process of die quenching, the deformation trend of the gear had changed many times, and the key deformation and phase transformation nodes were extracted for analysis, as shown in Figure 12. Under the influence of the temperature field, the gear produced shrinkage deformations and no phase transformation occurred within 10 s of quenching. During 10 s to 20 s, the martensite transformation occurred in the subsurface layer of the gear, and the expansion deformation caused by phase transformation was greater than the shrinkage deformation caused by temperature. Constrained by the die and load, the outer tooth top of the gear was warped and deformed. From 20 s to 46 s, as the temperature decreased, the core of the gear gradually completed the martensite transformation. The amount of martensite transformation in the inner part of the gear was more than that in the outer part, resulting in a warping deformation at the inner tooth top of the gear. At this time, the overall deformation of the gear showed an expansion trend compared with 20 s. The martensite transformation was completed in the surface layer after quenching for 50 s to 200 s. There was more martensite transformation in the inner part of the surface layer, resulting in a greater expansion deformation. At this time, there was no temperature difference between the inside and outside of the gear, and the structure transformation was essentially completed. Due to the large strength and stiffness of the gear, the pressure applied by the upper die was not enough to suppress and eliminate the deformation, and the warping of the inner tooth top was kept in the subsequent quenching process. After quenching, the gear expanded compared to the initial state.

**Figure 13:**
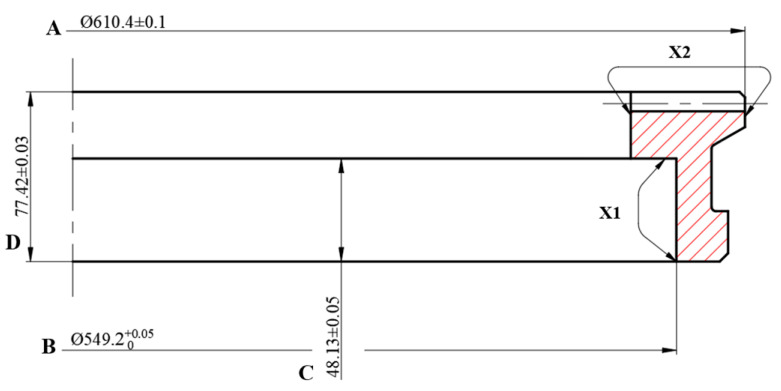
Measurement position of the face gear deformation (X1, X2: Carburizing area).

**Figure 14:**
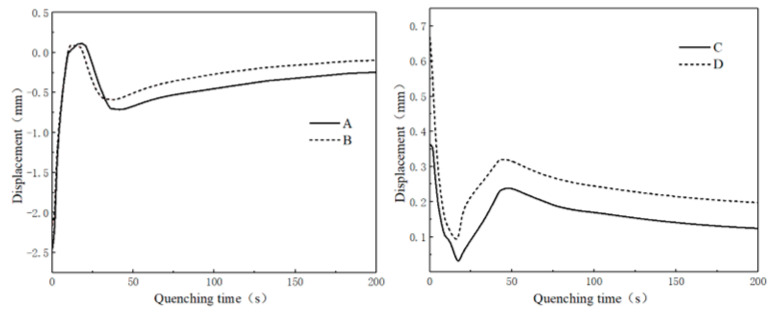
Axial and radial deformation of the gear during quenching for 200 s.

Four key positions were selected for deformation analysis: A and B represented radial displacement, while C and D represented axial displacement, as shown in Figure 13. The displacement changes at the key positions of the gear within 200 s of the quenching process are shown in Figure 14. The axial displacement and radial displacement both decreased in the initial stage of quenching, and the phase transformation began to occur at about 10 s, leading to increases in the displacements. The shrinkage deformation was greater than the expansion deformation at about 50 s, and the axial and radial displacements gradually decreased. Due to the limitation of the inner ring die, the radial displacement of B was less than that of A. Influenced by the warp of the tooth tip, the axial displacement of D located at the top of the tooth was larger than that of C.

### 3.2. Influence of Load on Gear Deformation

**Figure 15:**
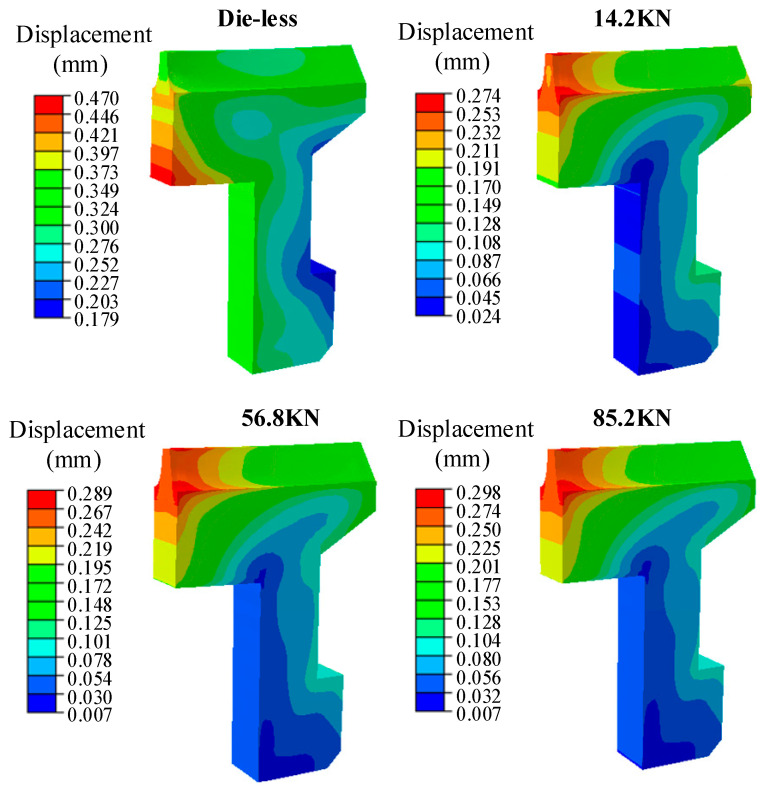
Deformation of Gear After Quenching Under Different Loads.

The load design of the die quenching is based mainly on experience and experiments. In order to minimize the distortion during the quenching, the load applied by the press should match the resistance required for anti-distortion. Simulation of the gear deformation trend under a variable force can reduce the number of experiments required to achieve the expected process results [18]. The load of face gear press’ was set as 14.2 kN, 56.8 kN, 71 KN, and 85.2 kN, and die-less was used as the deformation control group. The deformation cloud diagram of the gear after quenching is shown in Figure 15. The maximum deformation position of the die-less quenched gear was at the inner rib, the maximum deformation was 0.47 mm, and the minimum deformation position was 0.179 mm at the outer rib. The deformation at the top of the tooth was 0.36 mm. Constrained by the inner ring die and the upper die, the maximum deformation position of the die quenching of the gear was concentrated at the inner tooth top, and the minimum deformation position was at the inner rib. The maximum deformation at the inner tooth top increased with the gradual increase of the upper die load, and the top deformation and the minimum deformation of the gear approached a fixed value with the increase of the upper die load.

**Figure 16:**
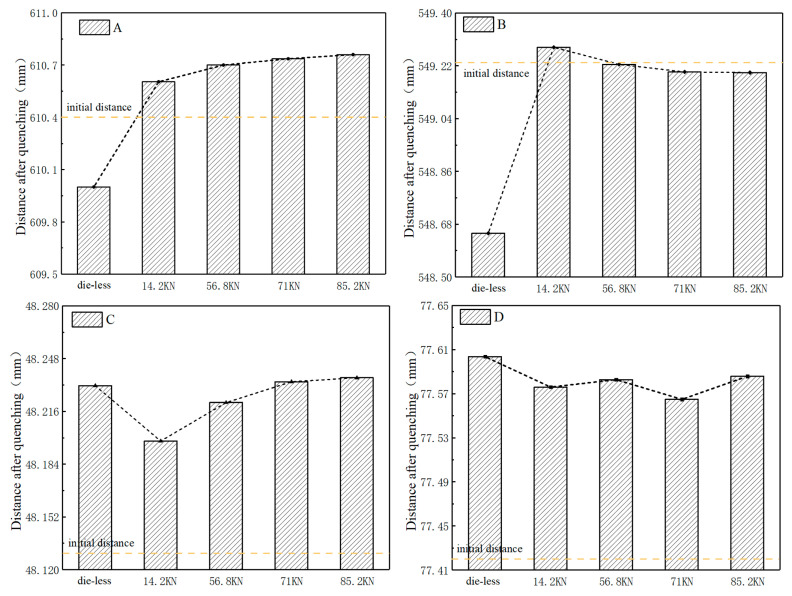
Deformation of gears in key positions (as shown in Figure 13A–D) under different loads.

Figure 16 shows the correlation law between the dimensions of the four key positions of the gear and the loads. For positions A and B the diameters decreased without the die constraint. Under the press load, the diameter of position A increased compared with the initial value, and with the increase of the load the diameter of position A gradually increased and tended to be stable. When the press load was 14.2 kN, the diameter at position B was larger than the initial diameter. With the increase of the load, the diameter at position B was gradually smaller than the initial value and tended to be stable. The diameters that changed at positions A and B showed that under the load of the upper die, the outer tooth top expanded outward and the rib shrunk inward. The inner ring die could effectively prevent the shrinkage deformation of the gear rib plate. For the axial dimensions at C and D, the dimensions were larger than the initial dimensions with or without the die, and the gear teeth were warped in the axial direction. The dimension at C increased with the increase of the load and tended to be stable gradually. The size of C could be effectively reduced without the die under a low load. With the increase of the load, the size of C gradually increased. Under the load, the top of the tooth changed into a saddle shape. During loading, the dimension of D was smaller than that without the die. Reasonable selections of the press load could effectively control the dimensions of D. The above analysis results showed that the die could effectively control the deformation of the face gear. The deformation of the gear by different loads was related to the position of the gear. In actual production, the load of the die should be reasonably selected according to the size requirements of each position.

## 4. Experimental Verification of Die Quenching

**Figure 17:**
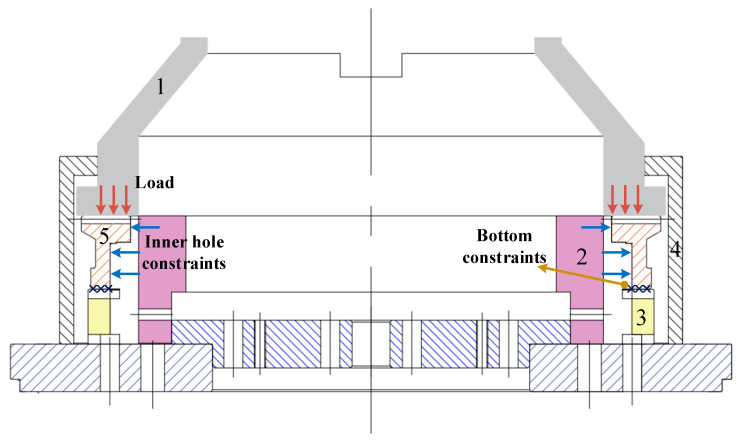
Assembly of the face gear quenching press: 1. upper die assembly; 2. inner hole support ring; 3. lower die assembly; 4. oil sealing ring; 5. The face gear.

**Figure 18:**
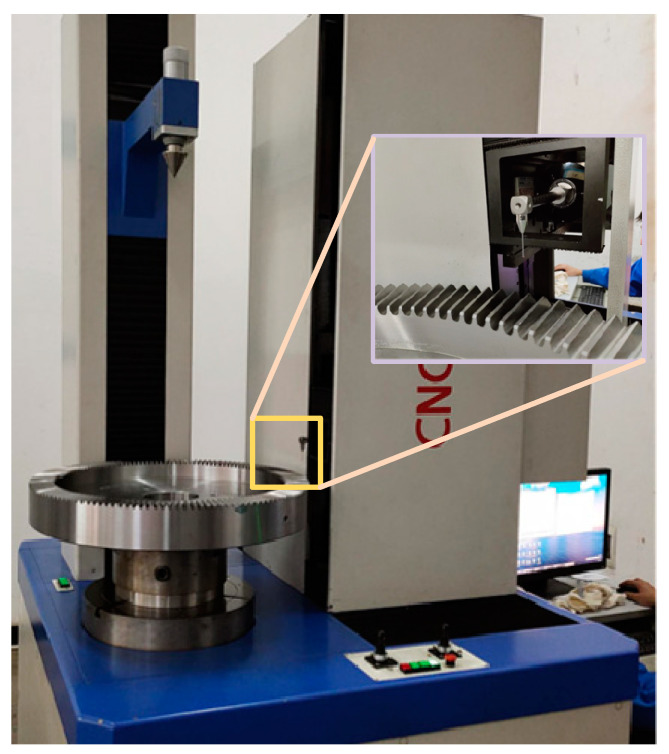
L65G high-precision gear, measuring center-measuring surface gear coordinates.

For the above simulation model parameters, the corresponding verification tests were carried out. Based on the heat treatment process route in Figure 5, the face gear was heated to 927 °C in the carburizing multipurpose furnace and held for 8 h for carburizing. The tooth surface and inner hub were carburized, and the other positions were protected by copper plating. After carburizing, the gear was cooled to room temperature under nitrogen protection. The gear was then heated to 621 °C for 4 h in an RJJ furnace, and cooled to room temperature with nitrogen after tempering. Before quenching, the gear was heated to 825 °C in an RJX furnace and held for 2 h to make the gear austenitic completely. An HEESS quenching press was used for load loading and unloading. After quenching, the gear was air-cooled to room temperature. In order to reduce the content of retained austenite and the internal stress of gears, a cryogenic treatment and tempering treatment were carried out after quenching [41]. The face gear press quenching equipment is shown in Figure 17. The inner hole and bottom surface of the gear were restricted by the die, and the top of the tooth was restricted by the upper die. The maximum load that the press could apply was 105 KN. The support force of the mandrel was 42 KN, the load of the upper die was 71 KN, and the pump power of quench oil flow was 1150 L/min. HVT-1000A image processing microhardness tester was used to measure the hardness of the gear. The test force was 500 gf, the load duration was 5 s, and the measurement multiple was 400 times. L65G gear measuring center was used to measure the gear coordinate. Compared with the ordinary coordinate, L65G can rotate the gear and the measurement method was more flexible. Figure 18 showed the process of the gear CMM measurement. The specific measurement positions are shown in Figure 13. The measurement results of the five groups of gear samples are shown in Table 5.

**Figure 19:**
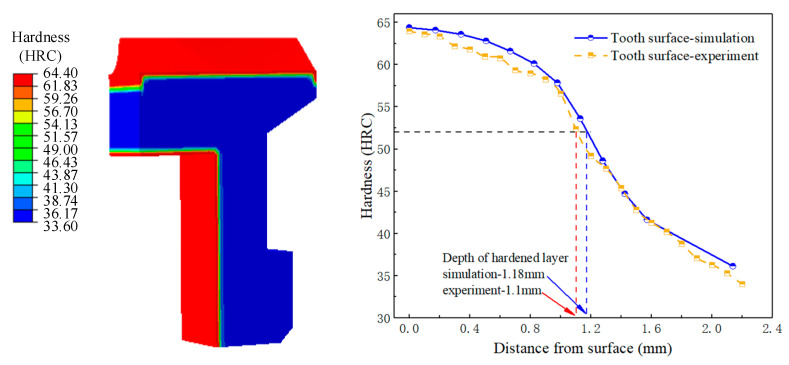
Comparison between simulation and experimental values of tooth surface hardness distribution.

The surface hardness gradient of the face gear sample was measured. The hardness distribution of the gear is shown in Figure 19. The simulated value of the surface hardness of the carburized area was 64.6HRC, and the core hardness was 33.6HRC, which was fairly consistent with the measurement results in Table 5. The hardness values of the tooth surface were measured every 100 mm along the layer depth direction, and the variation trend of the experimental and simulation values was the same. The hardness values gradually decreased with the increase of the layer depth. Taking 550 HV hardness as the standard, the effective hardened layer depth of simulation was 1.18 mm, the effective hardened layer depth of experiment was 1.1 mm, and the error between the two was 7.27%.

**Figure 20:**
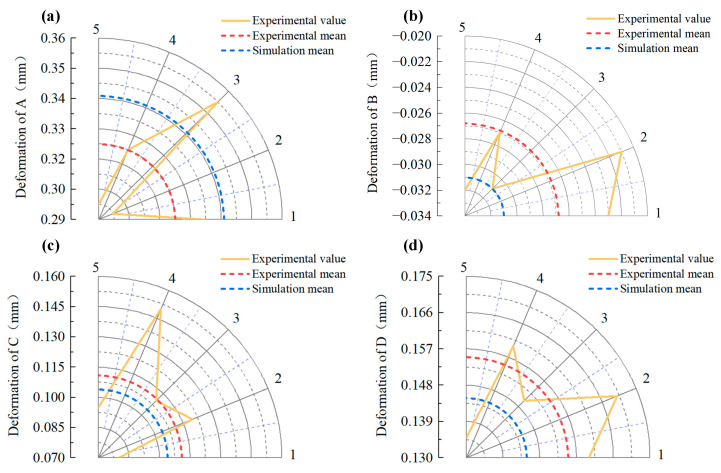
Comparison of quenching deformation results in key positions of gears (as shown in Figure 13): (**a**) A; (**b**) B; (**c**) C; (**d**) D.

Five groups of quenching deformation experimental values were used to compare and verify the simulation values of gear deformation, as shown in Figure 20. Due to the warping deformation of the gear, the expansion occurred at A, C, and D. In the quenching process, B was restricted by the inner ring die and finally turned into shrinkage deformation. The errors between the simulation values of deformation and the experimental mean values were less than 15% at four different locations, which verified that the established simulation model could effectively predict the actual deformation.

## 5. Conclusions

In this paper, based on the multi-field coupling theory of heat treatment, a finite analysis model for die quenching of 9310 face gear was established. FEA models were conducted to analyze the relationship between the die load constraint, the temperature-phase transformation field affected by carbon content, and the quenching deformation. The influence of stress on plastic deformation and phase change was not considered in the model, and the accuracy of the finite element model was verified by the die quenching experiment of the face gear.

After carburizing and quenching, the carbon content of the tooth surface was higher than that of tooth root. The hardness increased with the increase of the carbon content, and the calculated value of the hardness of the finite element calculation model was slightly larger than the experimental value, with a small error. The $M_{S}$ temperature was related to the carbon content. Based on the combined action of thermal deformation and phase transformation deformation, the gear produced shrinkage-expansion-shrinkage deformation and the top of the tooth was saddle-shaped at the end of quenching.

Compared with die-less quenching, die quenching can effectively reduce gear deformation. The maximum deformation of die-less quenching was concentrated at the inner rib, and the maximum deformation was 0.47 mm. The maximum deformation of die quenching was about 0.3 mm at the top of the tooth. The inner ring die could effectively control the shrinkage deformation of the rib. With the increase of the press load, the warping deformation of the tooth gradually increased and approached stability. The influence of different loads on the gear deformation was related to the specific position, and the press load should be reasonably selected according to the actual production requirements.

## Figures and Tables

**Table 1:** Parameters used in diffusive phase transformations.

Transformation	$\alpha_{1}$	$\beta_{1}$
Austenite to ferrite	0.360	3.468
Austenite to pearlite	0.360	3.468
Austenite to bainite	0.697	0.243

**Table 2:** Parameters used for non-diffusive phase transformations.

Transformation	$v_{m}$	$\alpha_{2}$	$\beta_{2}$	φ
Austenite to martensite	0.061	0.855	0.657	0.200

**Table 3:** Chemical composition (wt.%) of 9310 alloy steel.

Material Type	C	Mn	Ni	Cr	Cr	Mo	P	Si	Cu
9310	0.1	0.58	3.33	0.570	1.31	0.1	0.0026	0.20	0.015

**Table 4:** Thermal conductivity and specific heat of 9310 steel.

Phase	Thermal Conductivity/(J/m/°C)	Specific Heat/(J/kg/°C)
Austenite	16.0 + 1.3 × 10^−2^ T	365.0 + 0.2938 T
Martensite	25.0 + 3.0 × 10^−3^ T	450.0 + 0.3875 T

**Table 5:** Deformation and hardness measurement results of face gear.

Samples	A/mm	B/mm	C/mm	D/mm	Surface Hardness /HRC	Core Hardness /HRC
1	610.73	549.207	48.21	77.58	64	33.5
2	610.7	549.209	48.25	77.59	62.5	34
3	610.75	549.199	48.24	77.57	64	33.5
4	610.72	549.203	48.28	77.58	62.5	34
5	610.7	549.198	48.225	77.555	64	33.5
Average	610.72	549.203	48.241	77.575	63.4	33.7

## Data Availability

All data supporting the findings of this study are included within the article.
